# External validation of deep learning-based bone-age software: a preliminary study with real world data

**DOI:** 10.1038/s41598-022-05282-z

**Published:** 2022-01-24

**Authors:** Winnah Wu-in Lea, Suk-Joo Hong, Hyo-Kyoung Nam, Woo-Young Kang, Ze-Pa Yang, Eun-Jin Noh

**Affiliations:** 1 Department of Radiology, Guro Hospital, Korea University College of Medicine, Seoul, Republic of Korea; 2 Department of Pediatrics Endocrinology, Guro Hospital, Korea University College of Medicine, Seoul, Republic of Korea; 3 Smart Health-Care Center, Guro Hospital, Korea University College of Medicine, Seoul, Republic of Korea

**Keywords:** Endocrinology, Medical research

## Abstract

Artificial intelligence (AI) is increasingly being used in bone-age (BA) assessment due to its complicated and lengthy nature. We aimed to evaluate the clinical performance of a commercially available deep learning (DL)–based software for BA assessment using a real-world data. From Nov. 2018 to Feb. 2019, 474 children (35 boys, 439 girls, age 4–17 years) were enrolled. We compared the BA estimated by DL software (DL-BA) with that independently estimated by 3 reviewers (R1: Musculoskeletal radiologist, R2: Radiology resident, R3: Pediatric endocrinologist) using the traditional Greulich–Pyle atlas, then to his/her chronological age (CA). A paired *t*-test, Pearson’s correlation coefficient, Bland–Altman plot, mean absolute error (MAE) and root mean square error (RMSE) were used for the statistical analysis. The intraclass correlation coefficient (ICC) was used for inter-rater variation. There were significant differences between DL-BA and each reviewer’s BA (*P* < 0.025), but the correlation was good with one another (r = 0.983, *P* < 0.025). RMSE (MAE) values were 10.09 (7.21), 10.76 (7.88) and 13.06 (10.06) months between DL-BA and R1, R2, R3 BA. Compared with the CA, RMSE (MAE) values were 13.54 (11.06), 15.18 (12.11), 16.19 (12.78) and 19.53 (17.71) months for DL-BA, R1, R2, R3 BA, respectively. Bland–Altman plots revealed the software and reviewers’ tendency to overestimate the BA in general. ICC values between 3 reviewers were 0.97, 0.85 and 0.86, and the overall ICC value was 0.93. The BA estimated by DL-based software showed statistically similar, or even better performance than that of reviewers’ compared to the chronological age in the real world clinic.

## Introduction

Understanding the current status of skeletal maturity in children is important for evaluating developmental status and in detecting endocrinological abnormalities or metabolic disorders^1^. It also can be used to evaluate sexual maturity or to predict the final height of a child^2^.

The basis for skeletal maturation assessment relies on predictable changes in ossification centers over time. There have been numerous attempts to refine this assessment, which involves several different parts of the body, including the radius, ulna, teeth, and clavicle^3–5^. Among existing approaches, the two most commonly used standardized scoring methods for skeletal maturation are the Greulich–Pyle (GP) and Tanner–Whitehouse (TW) methods^6–8^. In the GP method, BA is estimated by comparing radiographs of the left hand and wrist with reference radiographs in the atlas. In the TW method, each bone of the left hand and wrist is given a score in comparison with a standard set of bones at different stages of maturation, and the total score is calculated to determine the BA. However, as both processes are rather time-consuming and the values tend to vary depending on the clinician’s experience, there have been optimization issues regarding their uses in BA assessment.

Recently, artificial intelligence (AI), especially deep learning (DL), is increasingly being used in a field of medicine**. **Deep learning is a subset of machine-learning technique that uses an algorithm to filter inputs through layers in order to learn to predict and classify data^9^. In 2018, a new DL–based automatic software system for BA estimation (BoneAge; *VUNO* Med, Seoul, South Korea) gained approval from the Korean Ministry of Food and Drug Safety, which was developed according to convolutional neural network. Its accuracy and efficiency were evaluated by the developers within the center, and the results suggested reliably accurate BA estimations and reduced reading times compared to manual GP methods^10^. However, for a deep learning based automatic software system to be used in clinical settings, a carefully designed external validation study is needed with datasets consisted of newly recruited patients or those from other institutions that exhibit similar characteristics to patients in a real-world setting^11^.

Therefore, we sought to evaluate the feasibility of DL software using the real-world data. We aimed to design a preliminary study using a dataset from the pediatric endocrinology clinic at our hospital.

## Materials and methods

### Study design

This is a retrospective study using patient’s hand x-ray and medical records in a large Korean tertiary teaching hospital. Ethical approval was obtained from and informed consent was waived by the institutional review board of the Korea University Guro Hospital. All methods were performed in accordance with the ethical standards of Helsinki Declaration.

### Patient selection

Children aged between 4 and 17 years who visited the pediatric endocrinology clinic at our hospital from November 2018 to February 2019 for a growth check-up involving left hand–wrist radiography were enrolled in this study. Although we planned to exclude those with bony abnormalities such as fracture, or congenital deformity, or those who had been diagnosed with any growth or endocrinological disorder, no such cases were presented. All the children were within the normal range of expected heights at their corresponding chronological ages (CA). Total 474 radiographs were subjected to assessment, comprising those of 35 boys and 439 girls. The mean age (± standard deviation) of included patients was 9.09 ± 1.68 years (range: 4–17 years).

### Image assessment

**Figure 1:**
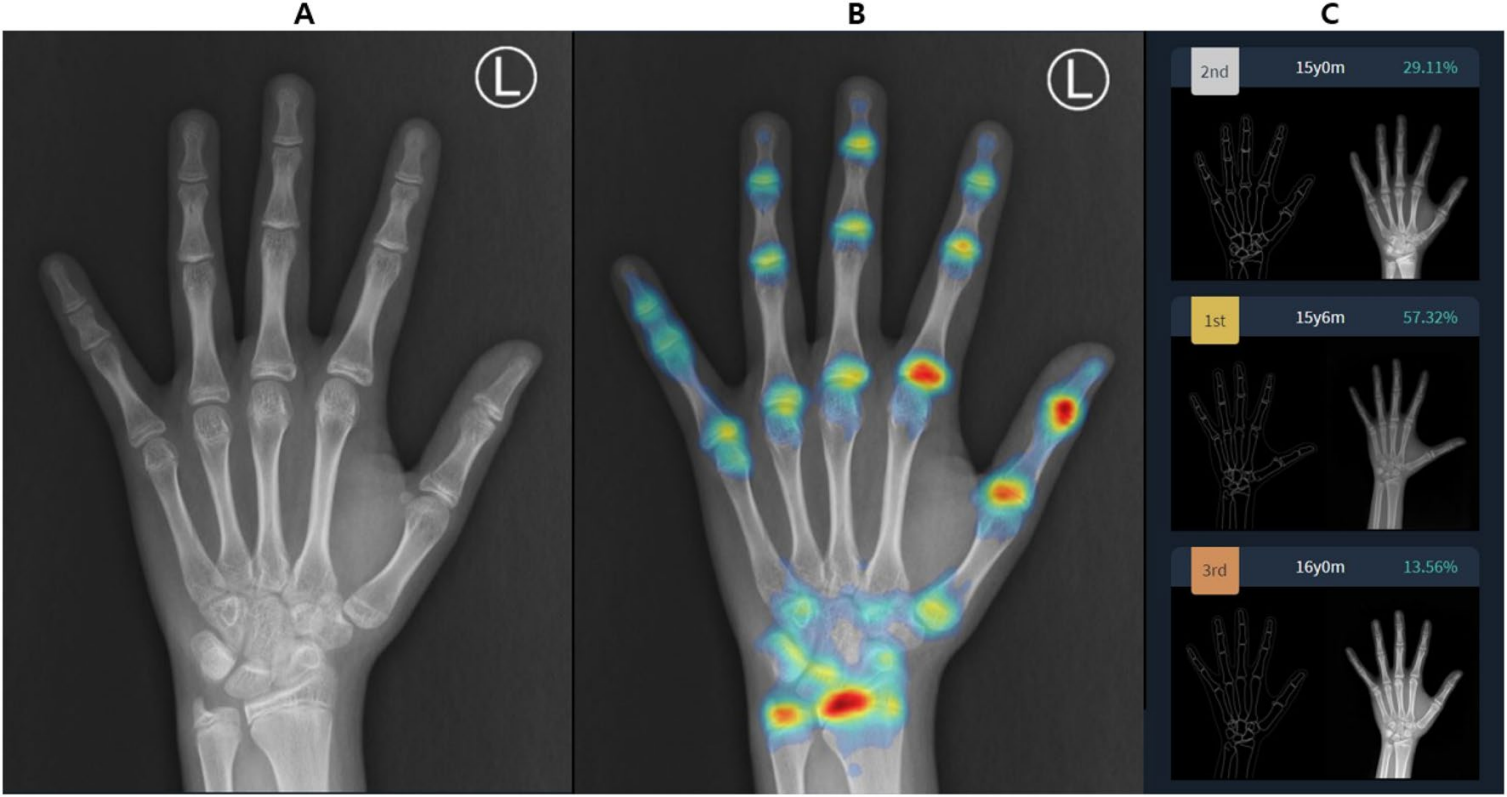
Screenshot of BA assessment by the software. Subject is a male patient with chronological age of 15 years and 3 month. (**A**) The software analyses the received left hand radiograph and (**B**) generates attention map using shape and density of each bone, then (**C**) displays estimated bone ages and each probability according to the attention map. First-rank bone age of this patient is 15 years and 6 months years with probability of 57.32%. The reference radiograph and outline-image depicting a specific shape of each bone are also shown in (**C**).

The BoneAge version 1.0.3 software program was used for automated BA assessment. Each patient’s image was sent to the DL software server in DICOM (Digital Imaging and Communications in Medicine) format assessed with the software, focusing on the shape and density of each bone. The software displayed as percentages the three most likely estimated BA values (i.e., first-, second-, and third-rank DL-BA) in order of probability **(**Fig. 1), and the first-rank BA value (i.e. the estimation with highest probability) was chosen as the DL estimation.

The same radiographs were subjected to assessment by two radiologists and one pediatric endocrinologist. Reviewer 1 (R1), a musculoskeletal radiologist, had 20 years of clinical experience in BA estimation. Reviewer 2 (R2), a second-year radiology resident, had no experience in BA estimation but participated in a training course involving the basics of BA estimation directed by R1 prior to their involvement in this study. Finally, reviewer 3 (R3) was a pediatric endocrinologist who had 10 years of clinical experience with BA estimation, mostly in the outpatient department. These three reviewers analyzed the radiographs independently using a traditional GP method, and the BA values were recorded in months. The readers were blinded to patient clinical information other than sex, including chronological age (CA) and results of automated BA assessment. The obtained BA values were then compared with the CA (in months) of each child, assuming that his/her estimated BAs correlates with his/her actual chronological age as the child is in normal range of expected heights.

### Statistical analysis

SPSS software (version 3.6.1; IBM Corporation, Armonk, NY, USA) was used to perform the statistical analysis. All variables were expressed as mean ± standard deviation A paired *t*-test was used to determine if there is a significant difference between the means of two groups. To see how well the two sets of data correlate with one another, Pearson’s correlation coefficient value was calculated. The mean absolute error (MAE) and root mean square error (RMSE) was calculated for the mean differences between the data to evaluate the agreement between the BA estimated by each reviewer and that of the software. A Bland–Altman plot was used to evaluate mean biases and 95% limit of agreements of estimated BA. Inter-rater variation was assessed using intraclass correlation coefficient (ICC). A *P* value less than 0.025 was considered statistically significant.

## Results

In this study, the estimated BAs ranged from 36 to 216 months for each reviewers and software. The range for CA was 44 to 219 months.

**Figure 2:**
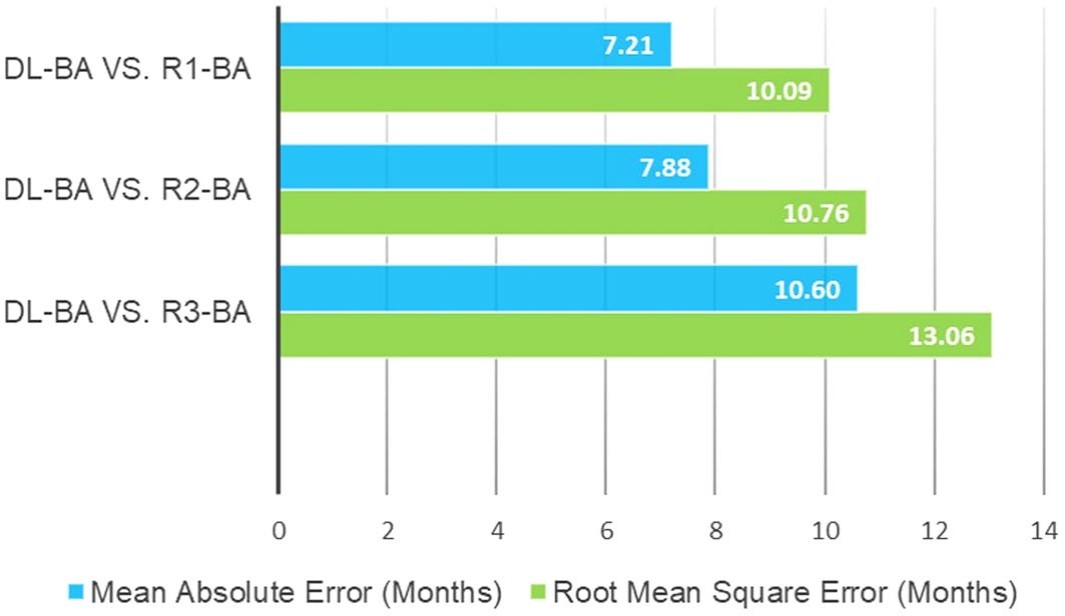
MAE and RMSE values between DL-estimated BA and each reviewer-estimated BA.

In the analysis with the DL-BA, the results showed that between R1-estimated BA (R1-BA) and DL-BA, paired *t*-test had *P* value of less than 0.025, which implies significant differences between them. Pearson’s correlation coefficient value was 0.98 also with *P* value less than 0.025, suggesting a strong correlation between them. The MAE value was 7.21 months and RMSE value was 10.09 months. The results were similar for between R2 and R3, and DL-BA; paired *t*-test had *P* value of less than 0.025 with Pearson’s correlation coefficient calculated as 0.98. The MAE and RMSE value were 7.88 and 10.76 months for R2, 10.60 and 13.06 months for R3, respectively (Table 1 and Fig. 2).

**Table 1:** Statistical analysis of DL-estimated and each reviewer-estimated BA, and chronological age.

	Mean absolute error (months)	Root mean square error (months)	Pearson's correlation coefficient*
CA vs. R1-BA	12.11	15.18	0.83
CA vs. R2-BA	12.78	16.19	0.80
CA vs. R3-BA	17.71	19.53	0.89
CA vs. DL-BA	11.06	13.54	0.87
DL-BA vs. R1-BA	7.21	10.09	0.98
DL-BA vs. R2-BA	7.88	10.76	0.98
DL-BA vs. R3-BA	10.60	13.06	0.98

*DL* deep learning, *CA* chronological age, *BA* bone age, *R* reviewer.

*All *P* < 0.025.

**Figure 3:**
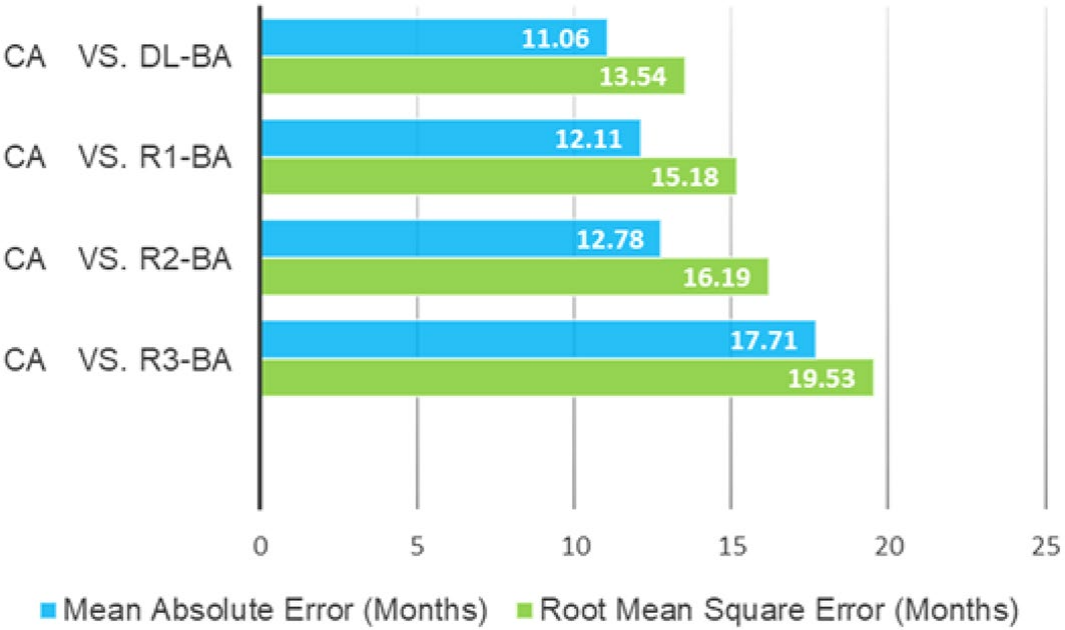
MAE and RMSE values between chronological age and each reviewer- and DL-estimated BA.

In the analysis with the CA, R1-BA demonstrated significant differences compared to the CA, with *P* value of *t*-test less than 0.025. Pearson’s correlation coefficient was calculated as 0.82, and the MAE and RMSE value were 12.11 and 15.18 months respectively. The other groups also demonstrated significant differences in each estimated BA compared to the CA with *P* value less than 0.025, with Pearson’s correlation coefficient ranging from 0.80 to 0.89, implying good correlation. The MAE and RMSE value were 12.78 and 16.19 months for R2, 17.71 and 19.53 months for R3, and 11.06 and 13.54 months for DL software (Table 1 and Fig. 3).

**Figure 4:**
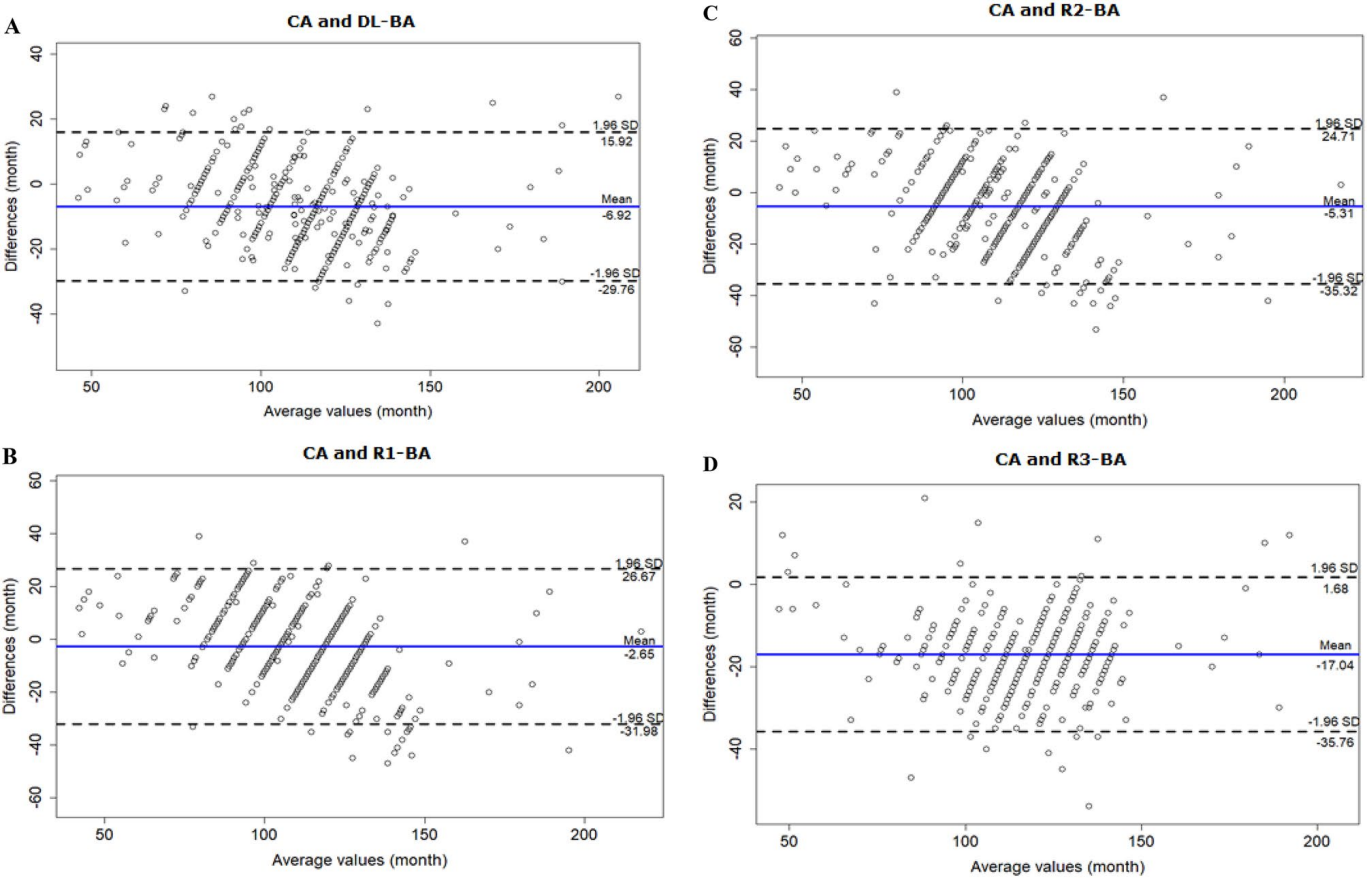
Bland–Altman plots for CA vs. DL and each reviewer-estimated BA. (**A**) Bland–Altman plot for CA and DL-estimated BA demonstrates DL’s tendency to overestimate BA, especially of children over the age of 120 months. (**B**) Bland–Altman plot for CA and R1-BA demonstrates R1’s tendency to overestimate BA, especially of children over the age of 110 months. (**C**) Bland–Altman plot for CA and R2-BA demonstrates R2’s tendency to overestimate BA, especially of children over the age of 110 months. (**D**) Bland–Altman plot for CA and R3-BA demonstrates R3’s tendency to overestimate BA, regardless of children’s age.

The Bland–Altman plot of agreement between the CA and DL-BA values showed a mean difference of −6.92 months, meaning DL tended to overestimate the BA in general. The cutoff value was 120 months, where DL tended to overestimate especially of patient over that age (Fig. 4A). According to the results, R1, R2 and R3 also tended to overestimate the BA. The cutoff value was 110 months for both R1 and R2 (Fig. 4B and C), while R3 tended to overestimate the BA regardless of patient’s age (Fig. 4D).

**Figure 5:**
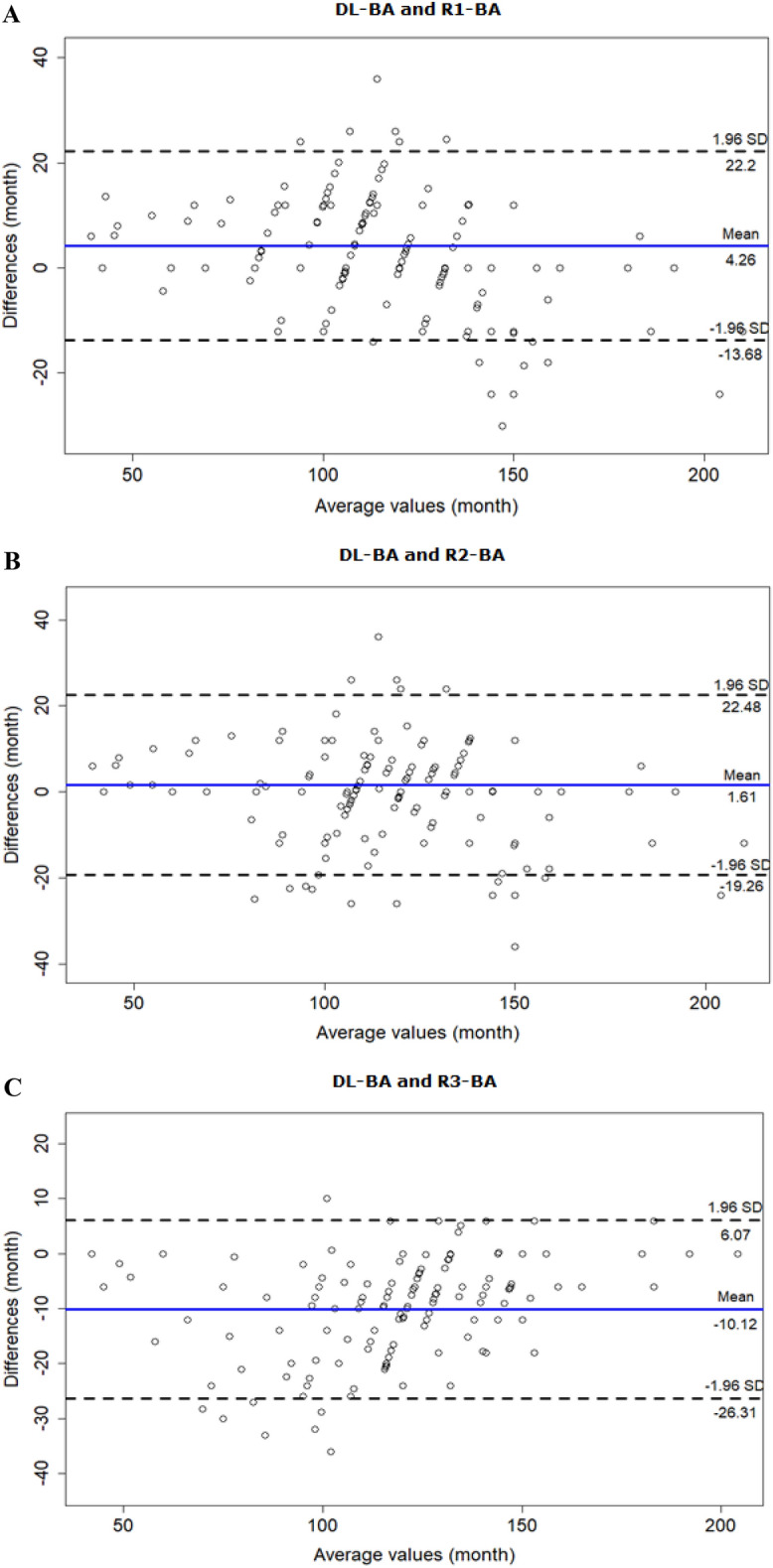
Bland–Altman plots for DL-estimated BA vs. each reviewer-estimated BA. (**A**) Bland–Altman plot for DL-BA and R1-BA demonstrates DL’s tendency to overestimate BA compare to R1, especially of children over the age of 110 months. (**B**) Bland–Altman plot for DL-BA and R2-BA demonstrates DL’s overall tendency to overestimate BA compare to R2. (**C**) Bland–Altman plot for DL-BA and R3-BA demonstrates R3’s tendency to overestimate BA compare to DL, especially of children below the age of 100 months.

 The agreement analysis was also performed between DL with each clinician. The results showed that DL tended to overestimate the BA compare to the clinicians except R3 (Fig. 5).

Inter-rater agreement was evaluated using the ICC. The values were 0.97 for R1 and R2, 0.85 for R1 and R3, 0.86 for R2 and R3. The overall ICC value of 0.93.

## Discussion

Due to its laborious nature and associated inter- and intra-observer variabilities, the need for automated BA measurement has always been interest of clinicians^12^. The first automated assessment technique named HANDX was developed in 1989 by Michael and Nelson, followed by PROI-based system by Pieka in 1991, computer-based skeletal aging scoring system (CASAS) by Tanner in 1994^13–15^. Subsequent to the rise of artificial intelligence in a field of medicine, many studies were performed on various AI-based bone age assessment solutions. BoneXpert is the first AI-based bone age assessment solution introduced in 2008. It uses feature extraction techniques and calculates bone age by analyzing the left-hand radiograph based on 13 bones^16^. It is now an established bone age assessment system and is widely used in Europe, with promising results of external validation^17^. Yet while it still adopts the machine-learning process of AI system, the software we evaluated, the BoneAge (V.1.0.3) is the first automated bone age assessment system that adopts the process of deep learning^10^.

External validation is important in verifying an overparameterized diagnostic or predictive classification model that is built with high-dimensional data such as deep learning algorithms to analyze radiologic images^18^. In this study, we performed an external validation to evaluate the feasibility of the BoneAge software program using a real-world clinical data set.

Internal validation of the software (BoneAge version 1.0.3) conducted by Kim et al. in 2017 resulted in an RMSE value of 7.2 months^10^. In our external validation, the RMSE values were 15.18, 16.19, 19.53, and 13.54 months for the musculoskeletal radiologist, radiology resident, pediatric endocrinologist, and DL software respectively, relative to the chronological age. This large differences in RMSE values between two studies may be due to the fact that Kim et al. used selected BAs from GP atlas as a reference determined by the consensus of two experienced radiologists, resulting the reference BA, reviewer and AI-estimated BA chosen from an equal set of data. In that case, if the software and reviewers’ performances were at best, a hand AP radiography with reference BA of 106 months would result in estimated BA of 106 months by the software and the reviewers, making the difference zero. However, our study used CA as a reference, a continuous variable which have a much larger range than GP atlas that is categorical. For example, one of the patients in our study had a chronological age of 101 months. Yet the closest estimated value the reviewers and the software can obtain was 94 or 106 months chosen from the atlas where the differences were still 7 or 5 months, respectively.

Our results demonstrated greater MAE and RMSE values between CA and reviewer-BA than those between CA and DL-BA (Average: 14.20 vs. 11.06 months, 16.97 vs 13.54 months). Although the values demonstrated significant differences in paired *t*-test, the results suggest that the DL software performed relatively better than all three reviewers, towards more accurate estimation of patient BA. On the other hands, if we exclude R3 from the analysis, a pediatrician who had the largest MAE and RMSE values among the reviewers, MAE is 12.45 (Average between the radiologists) vs. 11.06 months (DL) and RMSE 15.69 vs. 13.54 months. Furthermore, inter-reader agreement for R3 and R1 was somewhat weak compare to the one between R1 and R2 (0.85 vs. 0.97). R3’s relatively poor performance can be explained by the fact that the R3’s BA-estimation was done in real time at the outpatient clinic, which could have been very time-limiting. Therefore, our results enlighten the potential use of DL software in clinical settings by pediatricians as its performance is as accurate as, or even better than the radiologists’.

Our study had several limitations. First, the sample size was small and limited to Korean. As previous studies have reported the racial differences in growth patterns at certain ages^19^, further evaluations should be conducted in this context. Second, R3 was a pediatrician, not a radiologist. This could have led to biased results in evaluating the software’s performance, as Eitel, et al. pointed out that radiologists interpreted BAs differently than pediatric endocrinologists in the majority of images, interpreting as being older^20^. Nevertheless, considering we attempted to evaluate the feasibility of the software in actual clinical settings, with users in various fields of medicine and different degrees of experience in assessing BA, the results might have reflected the situation more accurately. Lastly, 92.6% of our patient cohort were female. As different patterns and stages of skeletal maturation have been reported between sexes^21^, the results might not be applicable to male patients. Further studies should include relatively similar percentages of both sexes.

## Conclusion

In this study, we investigated the feasibility of DL software for estimating BA by comparing the BA estimated by the software and three clinicians, the musculoskeletal radiologist, the second-year radiology resident, and the pediatric endocrinologist, to actual chronological age. The statistical analysis showed the software’s performance being as good as, or even better than the radiologists’. Therefore, we conclude that there is a promising future for the DL software to be used in actual clinical settings.

## References

[1] Zerin JM, Hernandez RJ (1991). Approach to skeletal maturation. Hand Clin.

[2] Martin DD, Wit JM, Hochberg Z (2011). The use of bone age in clinical practice - part 1. Horm Res Paediatr.

[3] Kumar V, Venkataraghavan K, Krishnan R, Patil K, Munoli K, Karthik S (2013). The relationship between dental age, bone age and chronological age in underweight children. J Pharm Bioallied Sci.

[4] Schmidt S, Mühler M, Schmeling A, Reisinger W, Schulz R (2007). Magnetic resonance imaging of the clavicular ossification. Int J Legal Med.

[5] Manzoor Mughal A, Hassan N, Ahmed A (2014). Bone age assessment methods: a critical review. Pak J Med Sci.

[6] Greulich WW, Pyle SI (1959). Radiographic atlas of skeletal development of the hand and wrist.

[7] Tanner JM (1962). Growth at adolescence : with a general consideration of the effects of hereditary and environmental factors upon growth and maturation from birth to maturity.

[8] Tanner JM (1983). Assessment of skeletal maturity and prediction of adult height (TW2 method).

[9] LeCun Y, Bengio Y, Hinton G (2015). Deep learning. Nature.

[10] Kim JR, Shim WH, Yoon HM, Hong SH, Lee JS, Cho YA, Kim S (2017). Computerized bone age estimation using deep learning based program: evaluation of the accuracy and efficiency. AJR Am J Roentgenol.

[11] Kim DW, Jang HY, Kim KW, Shin Y, Park SH (2019). Design characteristics of studies reporting the performance of artificial intelligence algorithms for diagnostic analysis of medical images: results from recently published papers. Korean J Radiol.

[12] Mansourvar M, Ismail MA, Herawan T, Raj RG, Kareem SA, Nasaruddin FH (2013). Automated bone age assessment: motivation, taxonomies, and challenges. Comput Math Methods Med.

[13] Michael DJ, Nelson AC (1989). HANDX: a model-based system for automatic segmentation of bones from digital hand radiographs. IEEE Trans Med Imaging.

[14] Pietka E, McNitt-Gray MF, Kuo ML, Huang HK (1991). Computer-assisted phalangeal analysis in skeletal age assessment. IEEE Trans Med Imaging.

[15] Tanner JM, Oshman D, Lindgren G, Grunbaum JA, Elsouki R, Labarthe D (1994). Reliability and validity of computer-assisted estimates of Tanner-Whitehouse skeletal maturity (CASAS): comparison with the manual method. Horm Res.

[16] Thodberg HH, Kreiborg S, Juul A, Pedersen KD (2009). The BoneXpert method for automated determination of skeletal maturity. IEEE Trans Med Imaging.

[17] Booz C, Yel I, Wichmann JL (2020). Artificial intelligence in bone age assessment: accuracy and efficiency of a novel fully automated algorithm compared to the Greulich-Pyle method. Eur Radiol Exp.

[18] Park SH, Han K (2018). Methodologic guide for evaluating clinical performance and effect of artificial intelligence technology for medical diagnosis and prediction. Radiology.

[19] Thodberg HH, Sävendahl L (2010). Validation and reference values of automated bone age determination for four ethnicities. Acad Radiol.

[20] Eitel KB, Eugster EA (2020). Differences in bone age readings between pediatric endocrinologists and radiologists. Endocr Pract.

[21] Cole TJ, Rousham EK, Hawley NL, Cameron N, Norris SA, Pettifor JM (2015). Ethnic and sex differences in skeletal maturation among the Birth to Twenty cohort in South Africa. Arch Dis Child.

